# Skeletal Muscle O-GlcNAc Transferase Action on Global Metabolism Is Partially Mediated Through Interleukin-15

**DOI:** 10.3389/fphys.2021.682052

**Published:** 2021-07-13

**Authors:** Morgan D. Zumbaugh, Con-Ning Yen, Jocelyn S. Bodmer, Hao Shi, David E. Gerrard

**Affiliations:** Department of Animal and Poultry Sciences, Virginia Polytechnic Institute and State University, Blacksburg, VA, United States

**Keywords:** O-GlcNAc signaling, interleukin-15, tissue cross-talk, insulin sensitivity, myokines

## Abstract

Besides its roles in locomotion and thermogenesis, skeletal muscle plays a significant role in global glucose metabolism and insulin sensitivity through complex nutrient sensing networks. Our previous work showed that the muscle-specific ablation of O-GlcNAc transferase (OGT) led to a lean phenotype through enhanced interleukin-15 (IL-15) expression. We also showed OGT epigenetically modified and repressed the *Il15* promoter. However, whether there is a causal relationship between OGT ablation-induced IL-15 secretion and the lean phenotype remains unknown. To address this question, we generated muscle specific OGT and interleukin-15 receptor alpha subunit (IL-15rα) double knockout mice (mDKO). Deletion of IL-15rα in skeletal muscle impaired IL-15 secretion. When fed with a high-fat diet, mDKO mice were no longer protected against HFD-induced obesity compared to wild-type mice. After 22 weeks of HFD feeding, mDKO mice had an intermediate body weight and glucose sensitivity compared to wild-type and OGT knockout mice. Taken together, these data suggest that OGT action is partially mediated by muscle IL-15 production and provides some clarity into how disrupting the O-GlcNAc nutrient signaling pathway leads to a lean phenotype. Further, our work suggests that interfering with the OGT-IL15 nutrient sensing axis may provide a new avenue for combating obesity and metabolic disorders.

## Introduction

Skeletal muscle is a major contributor to whole body metabolism and accounts for 70–90% of insulin-mediated glucose disposal (DeFronzo et al., 1981; Shulman et al., 1990; Watt et al., 2006; Bostrom et al., 2012; Funai et al., 2013; Song et al., 2013). Skeletal muscle maintains its mass, functionality, and metabolism through highly integrated signaling cascades that sense and respond to nutritional cues (Winder et al., 2000; Ebert et al., 2010; Ljubicic et al., 2011; Shi et al., 2018). The dynamic post-translational modification O-linked-β-D-N-acetylglucosamine (O-GlcNAc) serves as a widespread nutrient gauge by cycling on and off UDP-GlcNAc, an end product of the hexosamine biosynthetic pathway (HBP) that integrates carbohydrate, lipid, protein, energy, and nucleotide metabolism (Slawson et al., 2010; Hanover et al., 2012; Ruan et al., 2013). Specifically, O-GlcNAc transferase (OGT) and O-GlcNAcase (OGA) are responsible for the respective addition and removal of UDP-GlcNAc to modify target protein function according to fluctuating nutrient conditions (Issad et al., 2010; Ruan et al., 2012, 2014; Harwood and Hanover, 2014). For example, hyper-O-GlcNAcylation from prolonged exposure to PUGNAc, an OGA inhibitor, reduces skeletal muscle glucose disposal and attenuates insulin sensitivity (Arias et al., 2004). Additionally, chronic exposure of skeletal muscle to glucose, through GLUT4 overexpression, increases muscle UDP-GlcNAc levels and leads to the development of insulin resistance (Buse et al., 1996). Prolonged fatty acid or uridine exposure also increases muscle UDP-GlcNAc concentrations and attenuates muscle insulin sensitivity (Hawkins et al., 1997a,b).

Because the dysregulation of O-GlcNAcylation has been implicated in numerous metabolic disorders (Hanover et al., 2012; Ruan et al., 2013; Harwood and Hanover, 2014) and the modulation of skeletal muscle metabolism alters global metabolism (Izumiya et al., 2008; Meng et al., 2013), we investigated the role of skeletal muscle O-GlcNAcylation in whole body metabolic regulation. We found skeletal muscle O-GlcNAcylation plays an essential role in systemic energy homeostasis, and the deletion of skeletal muscle OGT (mKO) protects mice from high-fat diet-induced obesity and ameliorates whole-body insulin sensitivity (Shi et al., 2018).

The mKO phenotype bears a striking resemblance to mice overexpressing skeletal muscle specific interleukin-15 (IL-15). Overexpression of IL-15 in skeletal muscle also increases energy expenditure, increases expression of muscle oxidative enzymes, and protects mice from HFD induced obesity and insulin resistance; however, activity level and substrate utilization of these mice do not resemble mKO mice (Quinn et al., 2009, 2011, 2013). Indeed, mKO mice have a 5-fold increase in *Il15* expression in skeletal muscle and a 3-fold increase in IL-15 serum protein concentration. Therefore, we hypothesized OGT participates in the epigenetic regulation of *Il15* gene expression (Shi et al., 2018). In fact, EZH2, a key catalytic component of the polycomb repressive complex (PRC2), is a site of O-GlcNAcylation that may repress *Il15* expression through H3K27me3 methylation (Hanover et al., 2012; Chu et al., 2014; Lewis and Hanover, 2014). We found that OGT, EZH2, and H3K27me3 localized to the *Il15* promotor providing strong evidence OGT transcriptionally regulates *Il15* expression in skeletal muscle. However, we were unable to exclude that OGT deletion perturbed other cellular pathways that may contribute to the lean mKO phenotype. As a result, we aimed to determine if OGT action on global metabolism is attributed to IL-15 secretion using a genetic approach.

Secretion of IL-15 occurs in a unique manner that relies on the complexing of IL-15 and its high-affinity receptor interleukin-15 receptor alpha (IL-15rα) to increase the stability, secretion, and bioavailability of IL-15 (Rubinstein et al., 2006; Stoklasek et al., 2006). Co-expression of IL-15 and IL-15rα in human 293 cells increases secretion and stability of both molecules *in vitro* and *in vivo* (Bergamaschi et al., 2008). In skeletal muscle, the deletion of Il-15rα reduces IL-15 serum concentrations (O'Connell et al., 2015). Therefore, we generated skeletal muscle-specific OGT and IL-15rα double knockout mice (mDKO) to determine the contribution of IL-15 secretion to OGT action. After feeding a high-fat diet (HFD) for 22 weeks, mDKO mice exhibited an intermediate phenotype between mKO and wild-type (WT) mice corroborating our previous hypothesis that loss of OGT perturbs *Il15* expression and, at least partially, drives the mKO phenotype. Together, these findings suggest that IL-15 may be partially responsible for the lean phenotype observed in those mice lacking a functional *Ogt* gene in skeletal muscle.

## Materials and Methods

### Animals

Mice were generated and maintained at Virginia Polytechnic Institute and State University and all the experiments were approved by the Institutional Animal Care and Use Committee. Muscle specific *Ogt* and *Il15ra* deletion was accomplished by breeding *HSA*^Cre/+^; *Ogt*^LoxP/Y^; *Il15ra*^LoxP/LoxP^ males [Jax Mice strains: B6.Cg-Tg(ACTA1-cre)79Jme/J; B6.129-*Ogt^tm1Gwh^*/J; C57BL/6-*Il15ra^tm2.1Ama^*/J] with *Ogt*^loxP/loxP^; *Il15ra*^LoxP/LoxP^ females to generate *HSA*^Cre/+^; *Ogt*^LoxP/Y^; *Il15ra*^LoxP/LoxP^ (mDKO) and *HSA*^+/+^; *Ogt*^LoxP/Y^; *Il15ra*^LoxP/LoxP^ (WT) males. Mice were group housed with a 12-h light: 12-h dark cycle with *ad libitum* feed. At 4 weeks of age, male mice were fed with Teklad rodent diets (#TD.06414, Envigo) with 60% of calories coming from fat. The fatty acid profile (percentage of total fat) is as follows: 37% saturated, 47% monounsaturated, and 16% polyunsaturated.

### Body Composition

Body composition was determined after 22 weeks of HFD using a Bruker minispec LF90 TD-NMR analyzer (Bruker, Billerica, MA).

### Tissue Sample Collection

Mice were euthanized by carbon dioxide followed by cervical dislocation. *Tibialis anterior* (TA) and *Gastrocnemius* (GA) muscles and inguinal and epididymal white adipose tissue depots were collected and weighed.

### Quantitative RT-PCR

Directzol RNA Miniprep Kit (Zymo Research) was used to extract total RNA from the gastrocnemius muscle. High-Capacity cDNA Reverse Transcription Kit (Thermo Fisher Scientific) was used to perform reverse transcription. Fast SYBR Green Master Mix (Thermo Fisher Scientific) and 7500 Fast Real Time PCR System (Thermo Fisher Scientific) were used to carry out qPCR reaction. Quantification was performed using ΔΔCT method. Primers for OGT, IL-15rα, and IL-15 were as follows: OGT forward AAG AGG CAC GCA TTT TTG AC; OGT reverse ATG GGG TTG CAG TTC GAT AG; IL-15rα forward TGT CCA CCT CCC GTA TCT ATT; IL-15rα reverse AAA GCC AGA GTT ACA GAC ATA CC; IL-15 forward TCT TCA AAG CAC TGC CTC TTC; IL-15 reverse CCT CCT GTA GGC TGG TTA TCT.

### Serum IL-15 Quantification

Serum was collected from mice after 22 weeks of HFD feeding. Serum levels of IL-15/IL15rα complex were determined using an IL-15/IL-15R Complex Mouse ELISA kit (Invitrogen, Carlsbad, CA) according to the manufacturer’s instructions.

### Glucose and Insulin Tolerance Testing

Mice were fasted for 4 h before insulin tolerance testing (ITT). Bovine insulin (Sigma) was prepared in sterile saline and intraperitoneally injected at the dosage of 1 U/kg body weight. Blood glucose level was measured at 0, 15, 60, 90, and 120 min after insulin injection using an OneTouch Ultra2 test strip and a glucometer. For glucose tolerance testing (GTT), mice were fasted for 16 h before experiments were conducted. Glucose was intraperitoneally injected at 2 g/kg body weight, and blood collection and measurement were the same as ITT.

### Statistical Analysis

Results were normalized to WT and are presented as a fold change from the WT. Body weight, glucose tolerance testing, and insulin tolerance testing results are presented as means ± SEM. Data were analyzed using a student *t*-test. A value of *p* less than or equal to 0.05 was considered as significant. Comparisons were between WT and mDKO. Striped bars represent means from five mKO mice for reference and were not included in statistical analysis.

## Results

### Deletion of OGT and IL-15rα in Skeletal Muscle Increased Muscle *Il15* Gene Expression but Diminished Serum IL-15 Protein Concentrations

**Figure 1:**
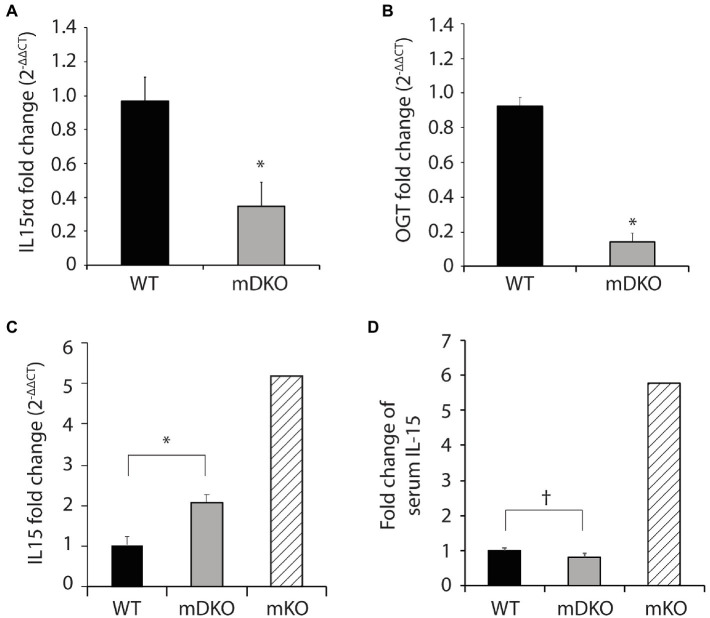
O-GlcNAc transferase (OGT) and interleukin-15 (IL-15)rα double knockout in skeletal muscle perturbed elevated IL-15 secretion. **(A–C)** Relative gene expression of **(A)** IL-15rα **(B)** OGT, and **(C)** IL-15 in the gastrocnemius muscles of WT and mDKO mice fed with a HFD for 22 weeks, *n* = 7. **(D)** Serum IL-15 levels in WT and mDKO mice fed a HFD for 22 weeks, *n* = 8. Values are normalized to WT means. ^*^p < 0.05, ^†^*p* < 0.08 when compared to WT. Values of striped bars are means from 5 muscle-specific OGT knockout mice (mKO) for reference.

To generate OGT and IL-15rα double knockout mice, we bred male mice harboring Cre-recombinase driven by expression of the human skeletal α-actin promotor (HSA-Cre mice) with *Ogt* and *Il15rα* floxed female mice to generate OGT and IL-15rα muscle-specific double knockout mice (mDKO). Gene expression of *Il15ra* (*p* = 0.011; Figure 1A) and *Ogt* (*p* < 0.0001; Figure 1B) were significantly reduced in mDKO muscle compared to WT, confirming the deletion of OGT and IL-15rα in skeletal muscle. We also measured *Il15* gene expression in mDKO muscle to determine if the simultaneous loss of OGT and IL-15rα recapitulated the dysregulated *Il15* expression observed in mKO mice. Indeed, *Il15* expression increased 2-fold in mDKO muscle (*p* = 0.004; Figure 1C) confirming OGT regulates *Il15* expression in mDKO skeletal muscle, albeit the concurrent deletion of IL-15rα abated the magnitude of increase observed in mKO mice (Figure 1C). In contrast, circulating IL-15 protein concentration tended to decrease in mDKO mice (*p* = 0.08; Figure 1D). While seemingly a trivial decrease, when referenced to circulating levels of IL-15 in mKO mice, it is apparent the loss of IL-15rα blunts IL-15 secretion from skeletal muscle. These data indicate the loss of IL-15rα impeded IL-15 secretion into circulation even though its gene expression was upregulated in skeletal muscle.

### Congruent Loss of OGT and IL-15rα Diminished the Protection Against HFD-Induced Obesity Observed in mKO Mice

**Figure 2:**
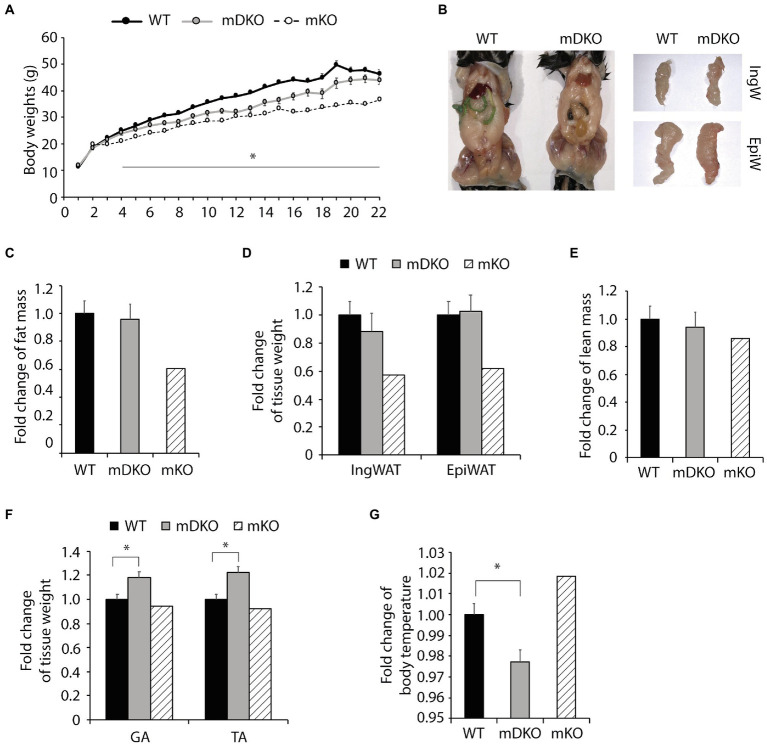
Loss of OGT and IL-15rα in skeletal muscle diminished the lean phenotype observed in OGT single knockout mice. **(A)** Weekly body weights of WT and mDKO mice during 22 weeks of HFD feeding. HFD feeding started at 4 weeks of age. Values are means ± SEM, *n* = 11–14. **(B)** Representative images comparing abdominal, inguinal (IngW), and epididymal (EpiW) white adipose tissue depots from WT and mDKO mice. **(C)** Fat composition and **(D)** weights of inguinal (IngW) and epididymal (EpiW) white adipose tissue depots of WT and mDKO mice after 22 weeks of HFD feeding, *n* = 8–11. **(E)** Lean composition and **(F)** muscle weights of gastrocnemius (GA) and tibialis anterior (TA) muscles of WT and mDKO mice after 22 weeks of HFD feeding, *n* = 8–11. **(G)** Rectal temperature of WT and mDKO mice, *n* = 8–11. Values are normalized to WT means. ^*^p < 0.05 when compared to WT. Values of striped bars are means from 5 muscle-specific OGT knockout mice (mKO) for reference.

To test whether OGT action is mediated through IL-15 in the context of obesity, we subjected mDKO mice to 22 weeks of a HFD. Body weights of mDKO mice were reduced compared to WT mice after 4 weeks of HFD feeding; however, mDKO body weights appeared to be greater than muscle-specific OGT single knockout (mKO) mice (Figure 2A). It appeared that mDKO mice had similar fat depots to WT mice (Figure 2B). Indeed, there were no significant differences in total fat mass (Figure 2C), inguinal white adipose tissue mass (IngW; Figure 2D), or epidydimal white adipose tissue mass (EpiW; Figure 2D) between WT and mDKO mice after 22 weeks of HFD feeding, although both WT and mDKO mice appeared to have greater fat accumulation than mKO mice (Figure 2C). Surprisingly, mDKO gastrocnemius (GA; *p* = 0.017) and tibialis anterior (TA; *p* = 0.003) weights increased compared to WT mice (Figure 2F), although total lean mass did not differ (Figure 2E). Previously, we reported mKO mice had a higher body temperature that corresponded to an increase in energy expenditure in mKO mice (Shi et al., 2018). Body temperatures of mDKO mice were lower than WT mice (*p* = 0.012; Figure 2G) suggesting decreased circulating levels of IL-15 may reverse the enhanced energy expenditure observed in mKO mice. Collectively, these data indicate the higher energy expenditure and leaner phenotype in mKO mice may be partially mediated through IL-15, as the decreased circulating IL-15 levels in mDKO mice negated this phenotype.

### Mice Lacking OGT and IL-15 Exhibited Glucose Intolerance After HFD Feeding

**Figure 3:**
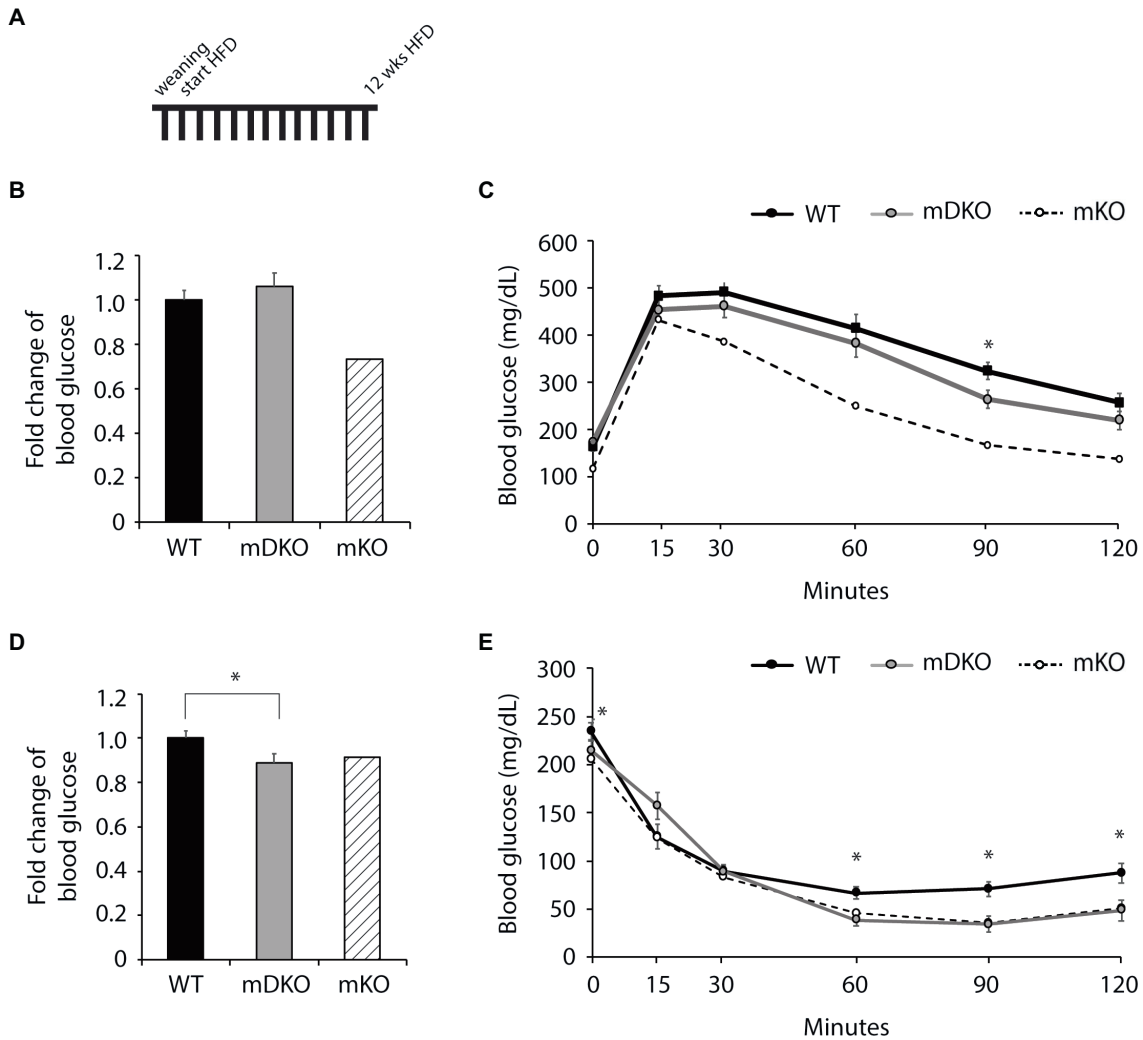
Glucose and insulin tolerance testing after 12 weeks of HFD feeding. **(A)** Schematic of HFD-feeding. **(B)** Resting blood glucose levels after 16 h of fasting in WT and mDKO mice. Values are normalized to WT means. **(C)** Glucose tolerance test of WT and mDKO mice. Values are means ± SEM. **(D)** Resting blood glucose levels after 4 h of fasting in WT and mDKO mice. Values are normalized to WT means. **(E)** Insulin tolerance test of WT and mDKO mice. Values are means ± SEM, *n* = 12–13. ^*^p < 0.05 when compared to WT. Values of striped bars are means from 5 muscle-specific OGT knockout mice (mKO) for reference.

To investigate whether the deletion of OGT and IL-15rα could negates the mKO protection against metabolic perturbations associated with obesity, we fed mice with a HFD. After 12 weeks of HFD feeding (Figure 3A), blood glucose levels were lower in mDKO mice after 4 h of fasting compared to WT (*p* = 0.054; Figure 3D), but there was no difference after 16 h of fasting (Figure 3B). Although WT and mDKO mice had similar blood glucose levels throughout the glucose tolerance testing (Figure 3C), their blood glucose levels appeared to be higher than mKO suggesting impaired glucose uptake. Insulin tolerance testing revealed lower blood glucose levels 60 min after insulin injection in mDKO mice compared to WT (Figure 3E).

**Figure 4:**
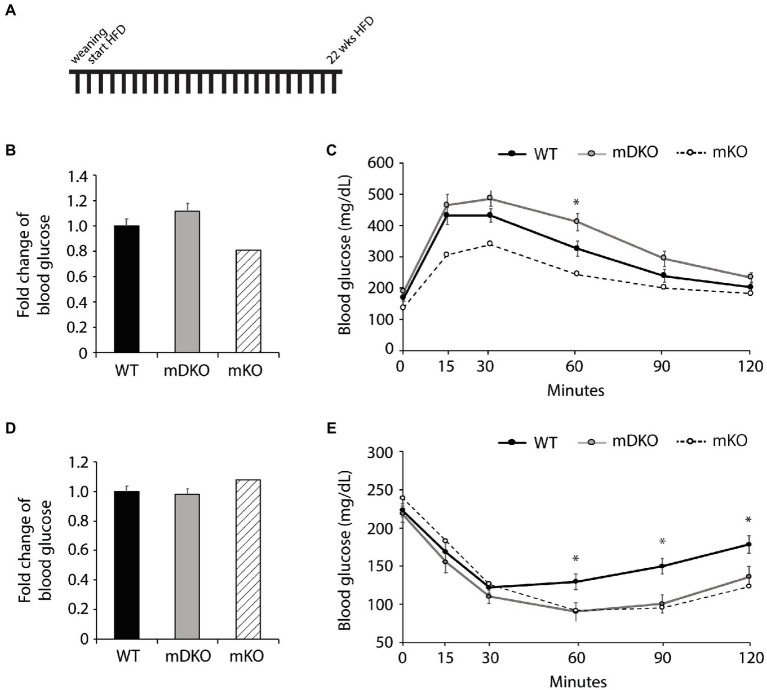
Glucose and insulin tolerance testing after 22 weeks of HFD feeding. **(A)** Schematic of HFD-feeding. **(B)** Resting blood glucose levels after 16 h of fasting in WT and mDKO mice. Values are normalized to WT means. **(C)** Glucose tolerance test of WT and mDKO mice. Values are means ± SEM. **(D)** Resting blood glucose levels after 4 h of fasting in WT and mDKO mice. Values are normalized to WT means. **(E)** Insulin tolerance test of WT and mDKO mice. Values are means ± SEM. *n* = 7–10. ^*^p < 0.05 when compared to WT. Values of striped bars are means from 5 muscle-specific OGT knockout mice (mKO) for reference.

After 22 weeks of HFD feeding (Figure 4A), there were no differences between WT and mDKO blood glucose levels after 4 h (Figure 4D) or 16 h (Figure 4B) of fasting. Blood glucose levels of WT and mDKO mice remained the same throughout glucose tolerance testing although mDKO mice had higher blood glucose levels than WT mice 60 min after the glucose injection (Figure 4C). Insulin tolerance testing at 22 weeks mirrored those from 12 weeks of HFD feeding (Figure 4E). These data indicate mDKO mice are susceptible to HFD-induced metabolic disorders but still retain some level of protection from the loss of OGT.

## Discussion

Previously, we showed the importance of skeletal muscle O-GlcNAcylation in the maintenance of whole-body metabolic homeostasis. Ablation of OGT, and thus O-GlcNAcylation, in skeletal muscle (mKO) resulted in increased energy expenditure, improved insulin sensitivity, protection against HFD-induced obesity, and dysregulation of muscle interleukin-15 (*Il15*) expression. We also showed the OGT-EZH2-H3K27me3 axis plays a critical role in the epigenetic regulation of *Il15* expression. However, it remains unknown if the increased IL-15 secretion alone caused the OGT phenotype. We hypothesized IL-15 secretion from muscle is a major contributor to the lean mKO phenotype because an oversecretion of IL-15 from skeletal muscle results in a similar protection against HFD-induced obesity and the onset of metabolic disorders. To test our hypothesis, we generated a model that permits OGT action on IL-15 in muscle but obstructs its secretion by ablating both OGT and interleukin-15 receptor alpha subunit (IL-15rα) in skeletal muscle (mDKO). We targeted IL-15rα rather than IL-15 because IL-15rα binds IL-15 to form a complex that increases the stability, secretion, and bioavailability of IL-15 (Rubinstein et al., 2006; Stoklasek et al., 2006; Bergamaschi et al., 2008). As such, the loss of IL-15rα reduces the stability and secretion of IL-15, which provides an ideal model to investigate OGT-mediated IL-15 action locally and globally. Indeed, mDKO muscle had a greater expression of IL-15 in muscle than WT but minimal circulating IL-15 levels. Although the decrease in mDKO IL-15 serum levels seems biologically irrelevant when compared to WT, the loss of IL-15rα drastically decreased circulating IL-15 when referenced to mKO. As such, it is a reasonable assumption that the loss of IL-15rα blunts IL-15 secretion from muscle, and thus its global action, regardless of local IL-15 expression. These findings correspond to muscle specific ablation of IL-15rα, which decreased IL-15 serum concentration approximately 4-fold in male mice (O’Connell et al., 2015) and support the notion of deleting IL-15rα in combination with OGT as a model to investigate the role of IL-15 secretion in the mKO lean phenotype.

Early reports of IL-15rα ablation implemented a ubiquitous deletion of IL-15rα that resulted in increased muscle oxidative enzymes and reduced adipose tissue mass, which initially suggested IL-15rα is not needed for effective IL-15 secretion from muscle and its subsequent modulation of global metabolism (Pistilli et al., 2011). However, muscle-specific ablation of IL-15rα did not decrease body weight or fat mass and had minimal impact on muscle oxidative enzymes (O’Connell et al., 2015). As such, reported differences in muscle and whole-body metabolism between the ubiquitous and muscle-specific knockout could be attributed to the global knockout lacking IL-15rα in the central nervous system. Specifically, IL-15 crosses the blood–brain barrier (Wu et al., 2010b) and modulation of IL-15/IL-15rα impacts sleep patterns (Kubota et al., 2001), anxiety behavior pathways (Wu et al., 2010a), and anti-depression pathways (Wu et al., 2011), which collectively has profound global impacts. The muscle-specific deletion of IL-15rα therefore defined its importance in modulating muscle and global metabolism (O'Connell et al., 2015), presumably through the stabilization and secretion of IL-15. To determine the role of IL-15 secretion from muscle in the mKO phenotype, we targeted both OGT, as previously reported, and IL-15rα, by breeding in mice harboring loxP sites flanking exons 2 and 3 of *Il15rα* (O'Connell et al., 2015), which respectively encode the sushi domain and linker region that play a role in the high affinity binding of IL-15 to IL-15rα (Dubois et al., 1999; Bouchaud et al., 2008). Using this model, we showed deleting IL-15rα in skeletal muscle localized IL-15 expression to muscle and impeded its secretion to negate the global effect of IL-15 overexpression in muscle. Although the decrease in mDKO serum IL-15 appears minimal compared to WT, the reduction is striking in relation to mKO IL-15 serum levels. Thus, it is reasonable to assume the loss of IL-15rα severely obstructs IL-15 stabilization and secretion in skeletal muscle also lacking OGT.

To determine whether the obstruction of IL-15 secretion in mDKO mice reverses the lean mKO phenotype, we fed mice a HFD for 22 weeks and found mDKO mice exhibited an intermediate phenotype between WT and mKO mice. These findings suggest that enhanced IL-15 circulation in mKO mice contributes, at least in part, to the lean mKO phenotype as ablation of both OGT and IL-15rα (mDKO) resulted in diminished protection from HFD-induce obesity. Although mDKO mice had greater similarities to WT mice than mKO mice, mDKO mice did not fully recapitulate the WT phenotype. However, this is not surprising given OGT has thousands of target proteins that are presumably responsible for the phenotypic discrepancies between mDKO, mKO, and WT mice. For example, there was no difference in fat mass and minimal differences in glucose disposal between WT and mDKO mice; however, mDKO mice were markedly different in insulin-stimulated glucose uptake 60 min after insulin administration. While circulating levels of IL-15 decreased in mDKO mice, IL-15 expression in muscle remained elevated, which may explain the discrepancies between mDKO GTT and ITT data given IL-15 is an established activator of the PI-3-kinase/Akt (Zhao and Huang, 2012; Lai et al., 2013) and Jak/STAT pathways (Johnston et al., 1995; Krolopp et al., 2016). As perturbations of these pathways have been implicated in the pathogenesis of metabolic diseases including insulin resistance (Dodington et al., 2018; Huang et al., 2018) and skeletal muscle accounts for 70–90% of global insulin-mediated glucose disposal, it is possible the increased local expression of IL-15 in mDKO muscle may improve glucose uptake upon insulin administration. Although GTT and ITT both test insulin sensitivity, GTT administers glucose to assess endogenous insulin response, whereas ITT administers insulin to stimulate tissue uptake of glucose. In our previous report, we found WT mice fed with a HFD develop insulin resistance and beta cells in the pancreas begin to “collapse” impairing beta cell response to injected glucose while mKO mice are protected. Given mDKO mice have minimal IL-15 serum concentrations, mimicking the WT phenotype, and have diminished insulin-mediated glucose disposal, it is plausible mKO protection against insulin insensitivity can be partially attributed to global IL-15 action. On the other hand, mDKO mice do not mimic the WT phenotype when insulin is administered, which can be attributed to greater local IL-15 expression in muscle. In fact, mKO muscle exhibited an increase in total protein abundance and Akt phosphorylation at T308 and S473 (Shi et al., 2018), which is involved in GLUT4 translocation. Indeed, GLUT4 expression increased in mKO muscle. Together mDKO GTT and ITT data suggest OGT action on IL-15 locally improves insulin-mediated glucose disposal when insulin is administered; however, obstruction of IL-15 global action abolishes beta cells protection from collapse. Although we were unable to elucidate the signaling mechanism responsible for opposing GTT and ITT results, it is reasonable to assume increased local IL-15 action in muscle plays a role in this phenomenon.

Further, activation of the PI-3-kinase/Akt pathway is also a potent stimulator of muscle hypertrophy and may provoke the increased GA and TA muscle mass in mDKO mice. Although mKO muscle had greater Akt phosphorylation and did not undergo hypertrophy, mKO mice also exhibited greater energy expenditure and body temperatures (Shi et al., 2018). Obstruction of IL-15 global action in mDKO mice prevented body temperature elevation, and presumably energy expenditure. A shift from catabolism to anabolism under conditions of increased local IL-15 expression in muscle may cultivate optimal conditions to provoke hypertrophy rather than burning substrates to fuel high energy expenditure. Therefore, it is plausible increased IL-15 expression in muscle may initiate a hypertrophic response that is facilitated by whole-body anabolism when IL-15 secretion from muscle is obstructed. These findings, to the best of our knowledge, are the first to investigate local IL-15 action on muscle independent from IL-15 global action.

In conclusion, our findings suggest IL-15 participates in muscle OGT global action, although it is not the sole contributor. However, this is not surprising that given OGT has thousands of known target proteins that regulate a wide range of cellular processes including protein localization, the cell cycle, signal transduction, mitochondrial bioenergetics, protein degradation, gene expression, and epigenetic regulation (Love and Hanover, 2005; Zachara et al., 2015). Even so, it is apparent that IL-15 is a major contributor to skeletal muscle OGT-mediated effects on local and global metabolism. In conclusion, our studies are the first to investigate the local action of OGT on IL-15 in muscle and the subsequent global effect of IL-15 on whole-body metabolism.

## Data Availability Statement

The original contributions presented in the study are included in the article material, further inquiries can be directed to the corresponding author.

## Ethics Statement

The animal study was reviewed and approved by Virginia Tech IACUC Committee.

## Author Contributions

MZ, HS, and DG designed the experiments and wrote the manuscript. MZ, CY, and JB conducted the experiments. All authors contributed to the article and approved the submitted version.

### Conflict of Interest

The authors declare that the research was conducted in the absence of any commercial or financial relationships that could be construed as a potential conflict of interest.

## References

[ref1] Arias E. B. Kim J. Cartee G. D. (2004). Prolonged incubation in PUGNAc results in increased protein O-linked glycosylation and insulin resistance in rat skeletal muscle. Diabetes 53, 921–930. 10.2337/diabetes.53.4.921, PMID: 15047606

[ref2] Bergamaschi C. Rosati M. Jalah R. Valentin A. Kulkarni V. Alicea C.. (2008). Intracellular interaction of interleukin-15 with its receptor alpha during production leads to mutual stabilization and increased bioactivity. J. Biol. Chem. 283, 4189–4199. 10.1074/jbc.M705725200, PMID: 18055460

[ref3] Bostrom P. Wu J. Jedrychowski M. P. Korde A. Ye L. Lo J. C.. (2012). A PGC1-alpha-dependent myokine that drives brown-fat-like development of white fat and thermogenesis. Nature 481, 463–468. 10.1038/nature10777, PMID: 22237023 PMC3522098

[ref4] Bouchaud G. Garrigue-Antar L. Sole V. Quemener A. Boublik Y. Mortier E.. (2008). The exon-3-encoded domain of IL-15ralpha contributes to IL-15 high-affinity binding and is crucial for the IL-15 antagonistic effect of soluble IL-15Ralpha. J. Mol. Biol. 382, 1–12. 10.1016/j.jmb.2008.07.019, PMID: 18656487

[ref5] Buse M. G. Robinson K. A. Marshall B. A. Mueckler M. (1996). Differential effects of GLUT1 or GLUT4 overexpression on hexosamine biosynthesis by muscles of transgenic mice. J. Biol. Chem. 271, 23197–23202. 10.1074/jbc.271.38.23197, PMID: 8798515

[ref6] Chu C. S. Lo P. W. Yeh Y. H. Hsu P. H. Peng S. H. Teng Y. C.. (2014). O-GlcNAcylation regulates EZH2 protein stability and function. Proc. Natl. Acad. Sci. U. S. A. 111, 1355–1360. 10.1073/pnas.1323226111, PMID: 24474760 PMC3910655

[ref7] DeFronzo R. A. Jacot E. Jequier E. Maeder E. Wahren J. Felber J. P. (1981). The effect of insulin on the disposal of intravenous glucose. Results from indirect calorimetry and hepatic and femoral venous catheterization. Diabetes 30, 1000–1007. 10.2337/diab.30.12.1000, PMID: 7030826

[ref8] Dodington D. W. Desai H. R. Woo M. (2018). JAK/STAT—emerging players in metabolism. Trends Endocrinol. Metab. 29, 55–65. 10.1016/j.tem.2017.11.001, PMID: 29191719

[ref9] Dubois S. Magrangeas F. Lehours P. Raher S. Bernard J. Boisteau O.. (1999). Natural splicing of exon 2 of human interleukin-15 receptor alpha-chain mRNA results in a shortened form with a distinct pattern of expression. J. Biol. Chem. 274, 26978–26984. 10.1074/jbc.274.38.26978, PMID: 10480910

[ref10] Ebert S. M. Monteys A. M. Fox D. K. Bongers K. S. Shields B. E. Malmberg S. E.. (2010). The transcription factor ATF4 promotes skeletal myofiber atrophy during fasting. Mol. Endocrinol. 24, 790–799. 10.1210/me.2009-0345, PMID: 20197309 PMC2852358

[ref11] Funai K. Song H. Yin L. Lodhi I. J. Wei X. Yoshino J.. (2013). Muscle lipogenesis balances insulin sensitivity and strength through calcium signaling. J. Clin. Invest. 123, 1229–1240. 10.1172/JCI65726, PMID: 23376793 PMC3582136

[ref12] Hanover J. A. Krause M. W. Love D. C. (2012). Bittersweet memories: linking metabolism to epigenetics through O-GlcNAcylation. Nat. Rev. Mol. Cell Biol. 13, 312–321. 10.1038/nrm3334, PMID: 22522719

[ref13] Harwood K. R. Hanover J. A. (2014). Nutrient-driven O-GlcNAc cycling—think globally but act locally. J. Cell Sci. 127, 1857–1867. 10.1242/jcs.113233, PMID: 24762810 PMC4004970

[ref14] Hawkins M. Angelov I. Liu R. Barzilai N. Rossetti L. (1997a). The tissue concentration of UDP-N-acetylglucosamine modulates the stimulatory effect of insulin on skeletal muscle glucose uptake. J. Biol. Chem. 272, 4889–4895. 10.1074/jbc.272.8.4889, PMID: 9030547

[ref15] Hawkins M. Barzilai N. Liu R. Hu M. Chen W. Rossetti L. (1997b). Role of the glucosamine pathway in fat-induced insulin resistance. J. Clin. Invest. 99, 2173–2182. 10.1172/JCI119390, PMID: 9151789 PMC508047

[ref16] Huang X. Liu G. Guo J. Su Z. (2018). The PI3K/AKT pathway in obesity and type 2 diabetes. Int. J. Biol. Sci. 14, 1483–1496. 10.7150/ijbs.27173, PMID: 30263000 PMC6158718

[ref17] Issad T. Masson E. Pagesy P. (2010). O-GlcNAc modification, insulin signaling and diabetic complications. Diabetes Metab. 36, 423–435. 10.1016/j.diabet.2010.09.001, PMID: 21074472

[ref18] Izumiya Y. Hopkins T. Morris C. Sato K. Zeng L. Viereck J.. (2008). Fast/glycolytic muscle fiber growth reduces fat mass and improves metabolic parameters in obese mice. Cell Metab. 7, 159–172. 10.1016/j.cmet.2007.11.003, PMID: 18249175 PMC2828690

[ref19] Johnston J. A. Bacon C. M. Finbloom D. S. Rees R. C. Kaplan D. Shibuya K.. (1995). Tyrosine phosphorylation and activation of STAT5, STAT3, and Janus kinases by interleukins 2 and 15. Proc. Natl. Acad. Sci. U. S. A. 92, 8705–8709. 10.1073/pnas.92.19.8705, PMID: 7568001 PMC41035

[ref20] Krolopp J. E. Thornton S. M. Abbott M. J. (2016). IL-15 activates the Jak3/STAT3 signaling pathway to mediate glucose uptake in skeletal muscle cells. Front. Physiol. 7:626. 10.3389/fphys.2016.00626, PMID: 28066259 PMC5167732

[ref21] Kubota T. Brown R. A. Fang J. Krueger J. M. (2001). Interleukin-15 and interleukin-2 enhance non-REM sleep in rabbits. Am. J. Phys. Regul. Integr. Comp. Phys. 281, R1004–R1012. 10.1152/ajpregu.2001.281.3.R1004, PMID: 11507019

[ref22] Lai Y. G. Hou M. S. Lo A. Huang S. T. Huang Y. W. Yang-Yen H. F.. (2013). IL-15 modulates the balance between Bcl-2 and Bim via a Jak3/1-PI3K-Akt-ERK pathway to promote CD8alphaalpha+ intestinal intraepithelial lymphocyte survival. Eur. J. Immunol. 43, 2305–2316. 10.1002/eji.201243026, PMID: 23754237

[ref23] Lewis B. A. Hanover J. A. (2014). O-GlcNAc and the epigenetic regulation of gene expression. J. Biol. Chem. 289, 34440–34448. 10.1074/jbc.R114.595439, PMID: 25336654 PMC4263851

[ref24] Ljubicic V. Miura P. Burt M. Boudreault L. Khogali S. Lunde J. A.. (2011). Chronic AMPK activation evokes the slow, oxidative myogenic program and triggers beneficial adaptations in mdx mouse skeletal muscle. Hum. Mol. Genet. 20, 3478–3493. 10.1093/hmg/ddr265, PMID: 21659335

[ref25] Love D. C. Hanover J. A. (2005). The hexosamine signaling pathway: deciphering the “O-GlcNAc code.” Sci. STKE 2005:re13. 10.1126/stke.3122005re13, PMID: 16317114

[ref26] Meng Z. X. Li S. Wang L. Ko H. J. Lee Y. Jung D. Y.. (2013). Baf60c drives glycolytic metabolism in the muscle and improves systemic glucose homeostasis through Deptor-mediated Akt activation. Nat. Med. 19, 640–645. 10.1038/nm.3144, PMID: 23563706 PMC3650110

[ref27] O'Connell G. Guo G. Stricker J. Quinn L. S. Ma A. Pistilli E. E. (2015). Muscle-specific deletion of exons 2 and 3 of the IL15RA gene in mice: effects on contractile properties of fast and slow muscles. J. Appl. Physiol. 118, 437–448. 10.1152/japplphysiol.00704.2014, PMID: 25505029

[ref28] Pistilli E. E. Bogdanovich S. Garton F. Yang N. Gulbin J. P. Conner J. D.. (2011). Loss of IL-15 receptor alpha alters the endurance, fatigability, and metabolic characteristics of mouse fast skeletal muscles. J. Clin. Invest. 121, 3120–3132. 10.1172/JCI44945, PMID: 21765213 PMC3148729

[ref29] Quinn L. S. Anderson B. G. Conner J. D. Pistilli E. E. Wolden-Hanson T. (2011). Overexpression of interleukin-15 in mice promotes resistance to diet-induced obesity, increased insulin sensitivity, and markers of oxidative skeletal muscle metabolism. Int. J. Interferon, Cytokine Mediat. Res. 3, 29–42. 10.2147/IJICMR.S19007, PMID: 28943758 PMC5605924

[ref30] Quinn L. S. Anderson B. G. Conner J. D. Wolden-Hanson T. (2013). IL-15 overexpression promotes endurance, oxidative energy metabolism, and muscle PPARdelta, SIRT1, PGC-1alpha, and PGC-1beta expression in male mice. Endocrinology 154, 232–245. 10.1210/en.2012-1773, PMID: 23161867 PMC3529369

[ref31] Quinn L. S. Anderson B. G. Strait-Bodey L. Stroud A. M. Argiles J. M. (2009). Oversecretion of interleukin-15 from skeletal muscle reduces adiposity. Am. J. Physiol. Endocrinol. Metab. 296, E191–E202. 10.1152/ajpendo.90506.2008, PMID: 19001550 PMC2636988

[ref32] Ruan H. B. Dietrich M. O. Liu Z. W. Zimmer M. R. Li M. D. Singh J. P.. (2014). O-GlcNAc transferase enables AgRP neurons to suppress browning of white fat. Cell 159, 306–317. 10.1016/j.cell.2014.09.010, PMID: 25303527 PMC4509746

[ref33] Ruan H. B. Han X. Li M. D. Singh J. P. Qian K. Azarhoush S.. (2012). O-GlcNAc transferase/host cell factor C1 complex regulates gluconeogenesis by modulating PGC-1alpha stability. Cell Metab. 16, 226–237. 10.1016/j.cmet.2012.07.006, PMID: 22883232 PMC3480732

[ref34] Ruan H. B. Singh J. P. Li M. D. Wu J. Yang X. (2013). Cracking the O-GlcNAc code in metabolism. Trends Endocrinol. Metab. 24, 301–309. 10.1016/j.tem.2013.02.002, PMID: 23647930 PMC3783028

[ref35] Rubinstein M. P. Kovar M. Purton J. F. Cho J. H. Boyman O. Surh C. D.. (2006). Converting IL-15 to a superagonist by binding to soluble IL-15R{alpha}. Proc. Natl. Acad. Sci. U. S. A. 103, 9166–9171. 10.1073/pnas.0600240103, PMID: 16757567 PMC1482584

[ref36] Shi H. Munk A. Nielsen T. S. Daughtry M. R. Larsson L. Li S.. (2018). Skeletal muscle O-GlcNAc transferase is important for muscle energy homeostasis and whole-body insulin sensitivity. Mol. Metab. 11, 160–177. 10.1016/j.molmet.2018.02.010, PMID: 29525407 PMC6001359

[ref37] Shulman G. I. Rothman D. L. Jue T. Stein P. DeFronzo R. A. Shulman R. G. (1990). Quantitation of muscle glycogen synthesis in normal subjects and subjects with non-insulin-dependent diabetes by 13C nuclear magnetic resonance spectroscopy. N. Engl. J. Med. 322, 223–228. 10.1056/NEJM199001253220403, PMID: 2403659

[ref38] Slawson C. Copeland R. J. Hart G. W. (2010). O-GlcNAc signaling: a metabolic link between diabetes and cancer? Trends Biochem. Sci. 35, 547–555. 10.1016/j.tibs.2010.04.005, PMID: 20466550 PMC2949529

[ref39] Song R. Peng W. Zhang Y. Lv F. Wu H. K. Guo J.. (2013). Central role of E3 ubiquitin ligase MG53 in insulin resistance and metabolic disorders. Nature 494, 375–379. 10.1038/nature11834, PMID: 23354051

[ref40] Stoklasek T. A. Schluns K. S. Lefrancois L. (2006). Combined IL-15/IL-15Ralpha immunotherapy maximizes IL-15 activity in vivo. J. Immunol. 177, 6072–6080. 10.4049/jimmunol.177.9.6072, PMID: 17056533 PMC2847275

[ref41] Watt M. J. Dzamko N. Thomas W. G. Rose-John S. Ernst M. Carling D.. (2006). CNTF reverses obesity-induced insulin resistance by activating skeletal muscle AMPK. Nat. Med. 12, 541–548. 10.1038/nm1383, PMID: 16604088

[ref42] Winder W. W. Holmes B. F. Rubink D. S. Jensen E. B. Chen M. Holloszy J. O. (2000). Activation of AMP-activated protein kinase increases mitochondrial enzymes in skeletal muscle. J. Appl. Physiol. 88, 2219–2226. 10.1152/jappl.2000.88.6.2219, PMID: 10846039

[ref43] Wu X. He Y. Hsuchou H. Kastin A. J. Rood J. C. Pan W. (2010a). Essential role of interleukin-15 receptor in normal anxiety behavior. Brain Behav. Immun. 24, 1340–1346. 10.1016/j.bbi.2010.06.012, PMID: 20600810 PMC2949491

[ref44] Wu X. Hsuchou H. Kastin A. J. He Y. Khan R. S. Stone K. P.. (2011). Interleukin-15 affects serotonin system and exerts antidepressive effects through IL15Ralpha receptor. Psychoneuroendocrinology 36, 266–278. 10.1016/j.psyneuen.2010.07.017, PMID: 20724079 PMC3015024

[ref45] Wu X. Pan W. Stone K. P. Zhang Y. Hsuchou H. Kastin A. J. (2010b). Expression and signaling of novel IL15Ralpha splicing variants in cerebral endothelial cells of the blood-brain barrier. J. Neurochem. 114, 122–129. 10.1111/j.1471-4159.2010.06729.x, PMID: 20374432 PMC2905142

[ref46] Zachara N. Akimoto Y. Hart G. W. (2015). “The O-GlcNAc modification,” in Essentials of Glycobiology. 3rd *Edn*. eds. Varki A. Cummings R. D. Esko J. D. Stanley P. Hart G. W. Aebi M.. (New York: Cold Spring Harbor), 239–251.

[ref47] Zhao H. Huang H. (2012). Functional capability of IL-15-Akt signaling in the denervated muscle. Cytokine 60, 608–615. 10.1016/j.cyto.2012.08.026, PMID: 23017227

